# GDF-15 and mtDNA Deletions Are Useful Biomarkers of Mitochondrial Dysfunction in Insulin Resistance and PCOS

**DOI:** 10.3390/ijms252010916

**Published:** 2024-10-10

**Authors:** Vera Varhegyi, Anna Modos, Domonkos Trager, Dora Gerszi, Eszter Maria Horvath, Miklos Sipos, Nandor Acs, Maria Judit Molnar, Szabolcs Varbiro, Aniko Gal

**Affiliations:** 1 Institute of Genomic Medicine and Rare Disorders, Semmelweis University, 1085 Budapest, Hungary; 2 Department of Obstetrics and Gynecology, Semmelweis University, 1085 Budapest, Hungary; 3 Department of Physiology, Semmelweis University, 1085 Budapest, Hungary; 4 Department of Obstetrics and Gynecology, Albert Szent-Györgyi Clinical Centre, University of Szeged, 6720 Szeged, Hungary; 5 Workgroup for Science Management, Doctoral School, Semmelweis University, 1085 Budapest, Hungary

**Keywords:** PCOS, insulin resistance, GDF-15, mitochondrial dysfunction, mtDNA deletion

## Abstract

There is no literature available about the growth differentiation factor-15 (GDF-15) biomarker in combination with mitochondrial DNA (mtDNA) deletions in insulin resistance (IR), and polycystic ovary syndrome (PCOS); however, it would be useful to achieve optimal metabolic status and improve pregnancy success. In this study, the role of GDF-15 and mtDNA deletions as biomarkers in the pathogenesis of IR and PCOS was investigated. In our study, 81 female patients who were treated for IR and/or PCOS and 41 healthy controls were included. GDF-15 levels in patients showed a marked increase compared to controls. Elevated GDF-15 levels were found in 12 patients; all of them had a BMI > 25 kg/m^2^, which is associated with reactive hyperinsulinemia. The presence of mitochondrial dysfunction was mainly observed in the IR-only subgroup. The increase in plasma levels of GDF-15 and the prevalence of mtDNA deletions is directly proportional to body mass index. The more marked metabolic abnormalities required more intensive drug therapy with a parallel increase in plasma GDF-15 levels. Elevated levels of GDF-15 and the presence of mitochondrial DNA deletions may be a consequence of carbohydrate metabolism disorders in patients and thus a predictor of the process of accelerated aging.

## 1. Introduction

Postponing childbearing until later in life means that more and more couples are facing infertility problems nowadays. Insulin resistance (IR) and polycystic ovary syndrome (PCOS) are common and treatable causes of infertility. Insulin resistance is one of the most prevalent metabolic disorders in the world to date, and is one of the major concerns of modern society with a prevalence of 15.5–46.5% [1,2]. Its clinical consequences, complications, and comorbidities such as type 2 diabetes mellitus (T2DM), obesity, metabolic syndrome, and PCOS affect a significant proportion of the global population. Insulin resistance has a high prevalence in women with polycystic ovary syndrome (PCOS). PCOS is an endocrine-dependent multifactorial disease characterized by ovulatory dysfunction, hyperandrogenism (hirsutism), and polycystic ovarian morphology, often accompanied by obesity and mild inflammation. PCOS affects 5 to 15% of women in their reproductive age. The diagnosis of PCOS is based on the Rotterdam criteria, where two of the three following features must be fulfilled: oligomenorrhea, hyperandrogenism (clinical or biochemical), and an ultrasonography image of polycystic ovaries. When PCOS is associated with type 2 diabetes then usually metformin treatment is suggested, which may possess geronto-protective properties. Studies suggest that model organisms treated with metformin show increased life expectancy and enhanced quality of life. El-Mir et al. reported that metformin selectively inhibits mitochondrial respiratory-chain complex 1 in rat hepatocytes [3]. This inhibition reduces NADH oxidation, causing a decreased proton gradient across the inner mitochondrial membrane and a lower oxygen consumption rate [3,4].

Mitochondrial dysfunction might also be involved in the pathogenesis of IR-PCOS. The ATP level and mitochondrial membrane potential are significantly lower in cells derived from patients with IR-PCOS; conversely, increased ROS levels are observed in cells from patients with IR-PCOS [5,6,7]. Additionally, insulin resistance is associated with decreased mitochondrial plasticity, which results in reduced insulin-stimulated mitochondrial activity [8]. This can lead to an increase in lipid metabolites, such as Acyl-CoA, DAG, and ceramides, which can further contribute to insulin resistance [9,10,11]. Mitochondrial plasticity, such as metabolic flexibility, is the limiting factor for in vivo ATP synthetic rates in insulin-resistant humans [8].

Mitochondrial biomarkers are molecules derived from the mitochondria and are mainly found in blood, urine, or cerebrospinal fluid, providing information about the function and current state of the mitochondria [12]. Based on recent publications, mitochondrial biomarkers can be divided into three main groups: (1) functional biomarkers in red blood cells; (2) biochemical markers in serum or plasma; and (3) DNA-based markers [13]. Among the DNA biomarkers, mtDNA copy number and mtDNA deletions are notable [14], and based on recent literature mtDNA deletions can be considered mitochondrial biomarkers in tumor diagnostics [15]. Among the plasma biomarkers, growth differentiation factor-15 (GDF-15) is currently the most commonly cited [16]. This is a stress-induced cytokine that is a member of the TGF-beta (transforming growth factor beta) superfamily [17]. It has been examined as a biomarker in several diseases, where it has been shown to be associated with the prognosis and progression of the pathological condition [10,11,12,13,18,19]. Among metabolic disorders, it is notable that elevated plasma GDF-15 levels have previously been found in T2DM patients [14,15]. Furthermore, a positive correlation was found between plasma GDF-15 concentration and insulin resistance, which was independent of age and BMI [20,21]. Recent literature has focused on identifying mitochondrial biomarkers that may play a role in the pathophysiology of PCOS and insulin resistance [22,23]. Mitochondria are vital for energy metabolism and their damage can lead to reduced insulin sensitivity in various tissues, including adipose tissue and skeletal muscle. Several mtDNA mutations and long non-coding RNAs (lncRNAs) were found to be part of an important regulatory mechanism in the etiology of PCOS [22]. Alterations in mitochondrial biomarkers, defined as cytochrome c (CC), acylcarnitine (AC), and citrate synthase (CS), have been used as reliable biomarkers of mitochondrial dysfunction, with a direct link to insulin resistance [23].

In the present study, we studied the correlation between GDF-15 levels, insulin resistance, and endocrine changes characteristic of PCOS-related infertility, and the possible influence of mtDNA deletion. We aimed to examine how the measurement of GDF-15 levels may influence the course of endocrinological and reproductive treatment.

## 2. Results

### 2.1. In IR Patients, Plasma GDF-15 Levels and the Presence of mtDNA Deletions Show a Significant Increase

**Figure 1:**
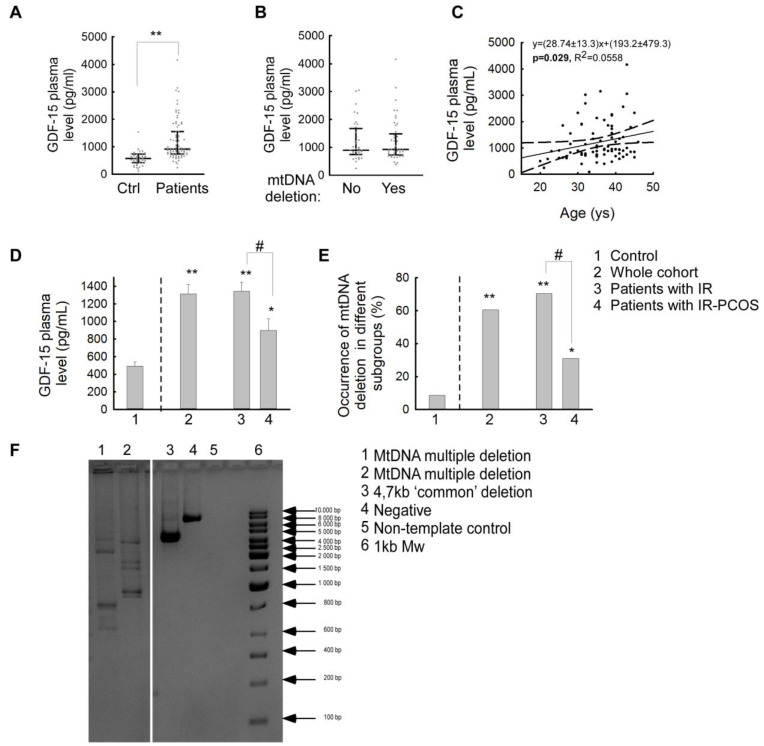
The plasma GDF-15 level pattern and the prevalence of mtDNA deletions in our investigated patients. (**A**) Median IQR plasma GDF-15 levels (expressed as pg/mL) of the whole patient group and the healthy controls, respectively (**: *p* < 0.001, Mann–Whitney U). (**B**) Median IQR plasma GDF-15 levels (expressed as pg/mL) in the investigated patients compared by the presence or absence of mtDNA deletions. (**C**) Correlation between the plasma GDF-15 levels and the age at examination (years) in the whole patient group (R^2^: 0.0557) (*p* < 0.05). (**D**) Mean plasma GDF-15 levels (expressed as pg/mL) in subgroups of patients with IR only, and both insulin resistance and PCOS (mean ± standard error of the mean (SEM); **: *p* < 0.001; *: *p* < 0.05; #: *p* < 0.05, Kruskall–Wallis Dunn). (**E**) Occurrence of mtDNA deletions in the control group (N = 41) (1), in the whole patient group (N = 81) (2), in patients with IR only (N = 62) (3), and in patients with both IR and PCOS (N = 19) (mean ± SEM; **: *p* < 0.001; *: *p* < 0.05; #: *p* < 0.05, Kruskall–Wallis Dunn). (**F**) Representative electrophoresis picture of mtDNA deletions, analyzed by long-range PCR (Mw: molecular weight marker (1000 kb).

The mean GDF-15 levels were significant increased in patients (mean GDF-15 plasma levels: 1213.58 ± 83.59 pg/mL (CI95: 1047.23–1379.93) compared to controls (mean GDF-15 plasma levels: 572.82 ± 82.76 pg/mL (CI95: 404.96–740.683) (*p* < 0.001) (Figure 1A). In the receiver operating characteristic (ROC) curve analysis based on the GDF-15 results of our patients and the healthy control group, the sensitivity and specificity of the test were found to be adequate (Figure S1). No significant differences in GDF-15 levels were found between the investigated cases with and without mtDNA deletions (mean GDF-15 plasma levels in patients without mtDNA deletions: 1187.582 ± 124.074 pg/mL (CI95: 1063.508–1311.656) (*p* = 0.014); mean GDF-15 plasma levels in patients with mtDNA deletions: 1230.566 ± 112.884 pg/mL (CI95: 1003.597–1457.353) (*p* = 0.877)), (Figure 1B) nor between single and multiple deletions (single deletion: 1337.776 ± 743.903 pg/mL (CI95: 966.627–1478.717); multiple deletions 1222.672 ± 789.865 pg/mL (CI95: 805.619–1869.932) (*p* = 0.552). In addition, the percentage of mtDNA deletions in patients with normal and high GDF-15 plasma levels showed a very similar distribution. In patients with normal GDF-15, mtDNA deletions were detectable in 57% of patients. Similarly, of patients with elevated GDF-15 plasma levels, 45% were mtDNA deletion-negative, while 55% had mtDNA deletions. In our investigated cases, GDF-15 plasma levels correlated with the age of the patients (R^2^ = 0.0558) (Figure 1C) similar to literature figures [24].

Next, we examined the GDF-15 levels in the different clinical subgroups. We found that in the IR-only subgroup, the mean plasma GDF-15 level was significantly higher than the average of the controls (mean GDF-15 plasma levels in IR patients: 1343.2 ± 105.9 pg/mL (CI95: 1131.021–1555.379) (*p* < 0.001)) (Figure 1E). In the IR-PCOS subgroup, GDF-15 levels were also significantly increased compared to healthy controls (mean GDF-15 plasma levels in IR-PCOS patients: 898.993 ± 131.065 pg/mL (CI95: 623.636–1174.35) (*p* = 0.003) (Figure 1E). It should be noted that when two subgroups of patients were compared (IR subgroup vs. IR-PCOS subgroup), a significant elevation in GDF-15 levels of the patients of the IR-only subgroup was also found (*p* = 0.011) (Figure 1E).

Interestingly, the prevalence of mtDNA deletion in our subgroups also shows a very similar pattern to the average plasma GDF-15 values. In our whole patient group, mtDNA deletions were detected in 61.5% of cases (Figure 1D) (single mtDNA deletions: 10.4%, multiple mtDNA deletions: 51.1%). The IR-only subgroup showed the highest prevalence of mtDNA deletion (70.3%), with 38 cases of multiple deletions and 7 cases of single deletion. In contrast, in the IR-PCOS subgroup, the percentage of mtDNA deletions was 31.5%, and only multiple deletions were detected. However, multiple deletion of mtDNA was also detected in 8.7% of the healthy control group (Figure 1D).

### 2.2. Strong Correlation of Elevated GDF-15 with Reactive Hyperinsulinemia

The association between plasma GDF-15 levels, the presence or absence of mtDNA deletions, and the most important parameters reflecting metabolic status at the time of sampling were investigated in each patient. These analyses could be performed in patients who had had a standard oral glucose tolerance (OGTT) test with 75 g of glucose solution.

**Figure 2:**
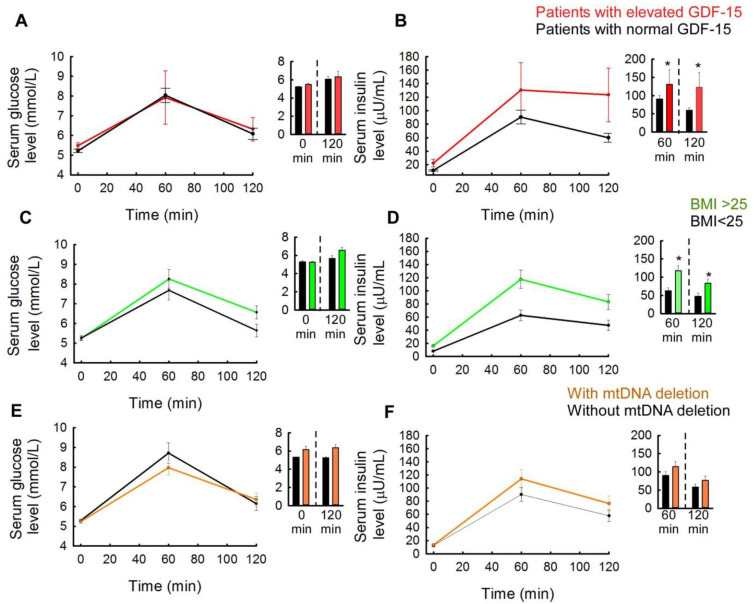
Correlations between the plasma levels of GDF-15 and the most important parameters that reflect the current metabolic status (serum glucose and insulin levels, body mass index), also in relation to the presence or absence of mtDNA deletions. (**A**) Serum glucose levels of the patients with elevated and normal GDF-15 levels, respectively, based on the results of the oral glucose tolerance test with 75 g of glucose (measured at minutes 0, 60, and 120 (patients with normal GDF-15 levels: N = 53; patients with elevated GDF-15 levels: N = 8)). (**B**) Serum insulin levels of the patients with elevated and normal GDF-15 levels, respectively, based on the results of the oral glucose tolerance test with 75 g of glucose (measured at minutes 0, 60, and 120 (patients with normal GDF-15 levels: N = 53; patients with elevated GDF-15 levels: N = 8)). (**C**) Serum glucose levels of the patients with high BMI and normal BMI levels, respectively, based on the results of the oral glucose tolerance test with 75 g of glucose (measured at minutes 0, 60, and 120 (patients with normal BMI (<25 kg/m^2^): N = 25; patients with elevated BMI (≥25 kg/m^2^): N = 36)). (**D**) Serum insulin levels of the patients with high BMI and normal BMI levels, respectively, based on the results of the oral glucose tolerance test with 75 g of glucose (measured at minutes 0, 60, and 120 (patients with normal BMI (<25 kg/m^2^): N = 25; patients with elevated BMI (≥25 kg/m^2^): N = 36)). (**E**) Serum glucose levels of the patients with mtDNA deletions (both single and multiple deletions) and without mtDNA deletions, respectively, based on the results of the oral glucose tolerance test with 75 g of glucose (measured at minutes 0, 60, and 120 (patients without mtDNA deletion: N = 23; patients with mtDNA deletion: N = 38)). (**F**) Serum insulin levels of the patients with mtDNA deletions (both single and multiple deletions) and without mtDNA deletions, respectively, based on the results of the oral glucose tolerance test with 75 g of glucose (measured at minutes 0, 60, and 120 (patients without mtDNA deletion: N = 23; patients with mtDNA deletion: N = 38)) (*: *p* < 0.05, paired *t*-test).

First, the serum glucose and insulin levels of the patients were measured from the standard oral glucose tolerance test (OGTT) with 75 g glucose at 0, 60, and 120 min and the slopes of the curves were compared in the different subgroups. When comparing patients with elevated and normal GDF-15 levels, no significant difference was found between the two subgroups for blood glucose values at minutes 0, 60, and 120 (Figure 2A). For serum insulin levels, the subgroups of elevated and normal plasma GDF-15 levels were significantly different. In particular, for insulin values at 60 and 120 min (patients with normal GDF-15 level: 60 min, 90.38 ± 58.63 µU/mL; 120 min, 59.87 ± 37.92 µU/mL; patients with elevated GDF-15 level: 60 min, 130.45 ± 90.54 µU/mL; 120 min, 88.67 ± 39.65 µU/mL) a significant increase was observed in the elevated plasma GDF-15 subgroup (*p* < 0.05) (Figure 2B). Patients with elevated plasma GDF-15 also had significantly higher HOMA index levels (patients with normal GDF-15 level: 2.9 ± 2.5 (CI95: 2.2–3.5); patients with elevated GDF-15 level: 4.4 ± 2.7 (CI95: 2.4–6.4) (*p* < 0.05)). Since patients with elevated GDF-15 levels are almost always associated with a high body mass index, it was considered necessary to perform the analysis with the patient group divided into different body mass index ranges to exclude the possibility that reactive hyperinsulinemia is associated with high GDF-15 levels and not with an elevated BMI. For the analysis, patients were divided into two groups based on body mass index: those with a BMI below 25 kg/m^2^ and those with a BMI above or equal to 25 kg/m^2^. In the subgroup of patients with elevated BMI, the mean glucose value at 120 min showed an increasing trend (*p* = 0.07); in patients with normal BMI (<25 kg/m^2^), the mean glucose value at 120 min was 5.64 ± 1.2 mmol/L, and in patients with elevated BMI (≥25 kg/m^2^) the value was 6.54 ± 1.66 mmol/L. However, there was no significant difference between the two subgroups at any of the sampling time points of the OGTT (Figure 2C).

Fasting mean insulin levels showed a significant difference (increase) in patients with high BMI (*p* < 0.05), similar to that observed in the subgroup of patients with elevated plasma GDF-15 levels (BMI < 25, 60 min: 62.62 ± 30.28 U/mL; 120 min: 47.41 ± 29.43 U/mL; elevated GDF-15, 60 min: 117.46 ± 69.53 U/mL; 120 min: 83.16 ± 37.03 U/mL). There was no significant difference in serum insulin levels at 60 and 120 min between the two groups (Figure 2D).

When 60 min insulin values were compared between the high BMI and elevated GDF-15 subgroups, the mean insulin values in the GDF-15 subgroup showed a slight increase, but this could not be confirmed by statistical tests (*p* = 0.933). Therefore, it is not clear whether the difference seen is due to the effect of elevated GDF-15 or to the associated elevated BMI values observed in patients (Figure 2B,D). Finally, two subgroups of patients were also formed based on the presence or absence of single or multiple mitochondrial DNA deletions (Figure 2E,F). Here, we found no significant difference in blood glucose levels and the 60 min glucose level was even lower in the mtDNA deletion subgroup than in those without mtDNA deletions (Figure 2E). In contrast, a slight increase in insulin values at the 60th and 120th minutes was observed in the subgroup with mtDNA deletions compared to the subgroup without mtDNA deletions (Figure 2F).

### 2.3. Strong Correlation of Elevated GDF-15 Levels with High Body Mass Index

**Figure 3:**
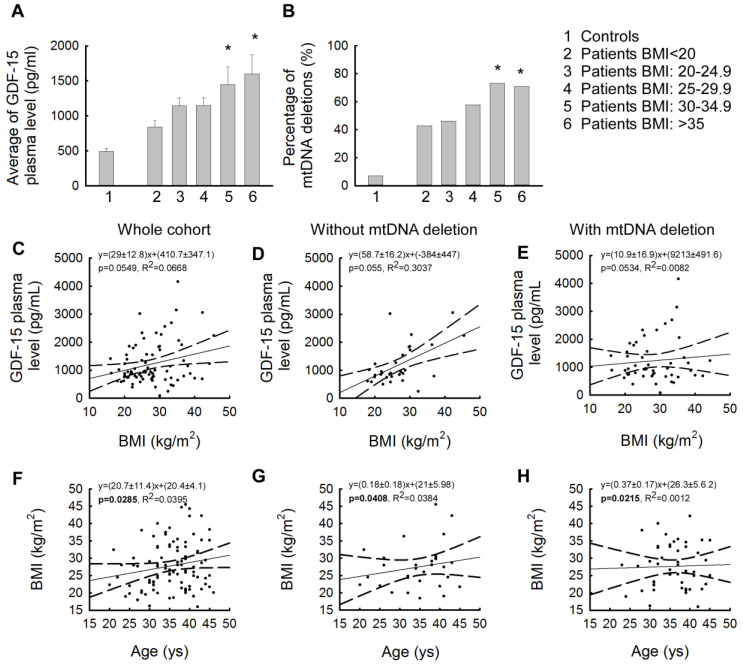
Correlations between the plasma levels of GDF-15 and the presence or absence of mtDNA deletions, and the BMI and the age at examination. (**A**) Mean + SEM plasma GDF-15 levels for different body mass index ranges compared to plasma GDF-15 levels in healthy controls (*: *p* < 0.05, Kruskall–Wallis Dunn). (**B**) The percentage of individuals with mtDNA deletions in different body mass index ranges in the patient group compared to the percentage of individuals with mtDNA deletions in healthy controls (*: *p* < 0.05, Kruskall–Wallis Dunn) (BMI subgroups: patients with BMI < 20 (N = 8); patients BMI: 20–24.9 kg/m^2^ (N = 25); patients with BMI: 25–29.9 kg/m^2^ (N = 22); patients with BMI: 30–34.9 kg/m^2^ (N = 13); and patients with BMI: >35 kg/m^2^ (N = 22). (**C**) Correlation between plasma GDF-15 levels and body mass index values in the whole patient group (R^2^ = 0.0668). (**D**) Correlation between plasma GDF-15 levels and body mass index values in the subgroup of patients without mtDNA deletions (R^2^ = 0.3071). (**E**) Correlation between plasma GDF-15 levels and body mass index values in the subgroup of patients with mtDNA deletions (R^2^ = 0.00882). (**F**) Correlation between body mass index values and age of examination in the whole patient group (R^2^ = 0.03395). (**G**) Correlation between body mass index values and age at examination in patients without mtDNA deletions (R^2^ = 0.04088). (**H**) Correlation between body mass index values and age of examination in patients with mtDNA deletions (R^2^ = 1.01012 × 10^−3^).

We also examined mean plasma GDF-15 levels by how plasma GDF-15 levels varied by body mass index range (<20 kg/m^2^; 20–24.9 kg/m^2^; 25–29.9 kg/m^2^; 30–34.9 kg/m^2^; >35 kg/m^2^) compared to healthy controls. While the mean GDF-15 levels in patients with BMI values below 20 kg/m^2^ were similar to those in the control group, the increase in plasma GDF-15 levels was observed in direct proportion to the increase in BMI values (Figure 3A). The largest increases were found in the two BMI categories for those with a BMI above 30 kg/m^2^. Patients with a BMI between 30 and 34.9 kg/m^2^ showed only an increasing trend compared to the control (the average GDF-15 level in controls: 572.82 ± 82.76 pg/mL; in patients with a BMI between 30 and 34.9 kg/m^2^: 1450.19 ± 250.95 pg/mL, *p* = 0.266), while a significant difference was found in the extreme obese subgroup (BMI: >35 kg/m^2^ (the average GDF-15 level in controls: 850.75 ± 132.45 pg/mL; in patients with a BMI of 30–34.9: 1600.69 ± 572.36 pg/mL, *p* < 0.05)). At the same time, the subgroups within the BMI ranges detailed above were also examined in terms of the percentage of mtDNA deletions that occurred in these subgroups, and this percentage of occurrence was also analyzed for the control group. We found that similar to the change in plasma GDF-15 values, as BMI increased, the prevalence of mtDNA deletions increased as well (Figure 3B). Similar to the GDF-15 values, high percentages of mtDNA deletions were also observed in patients with BMI >30 kg/m^2^, with a prevalence of mtDNA deletion of more than 70% (Figure 3B). In our investigated patients, an association between GDF-15 and BMI was detected (Figure 3C). The association of GDF-15 and BMI was significantly greater in mtDNA deletion-negative cases than in the subgroup having single or multiple mtDNA deletions (Figure 3D,E). In addition, we examined the linear regression of age and BMI (Figure 3F), which interestingly showed a weaker correlation in the presence of mitochondrial dysfunction than in the subgroup of patients without mtDNA deletions (Figure 3G,H).

The correlation between plasma GDF-15 levels and the age at examination was also investigated for the whole study group, and a comparison of the study group and control group by age was performed for plasma GDF-15 levels (Figure S2).

### 2.4. Association between Plasma GDF-15 Levels and Different Treatments for Insulin Resistance

**Figure 4:**
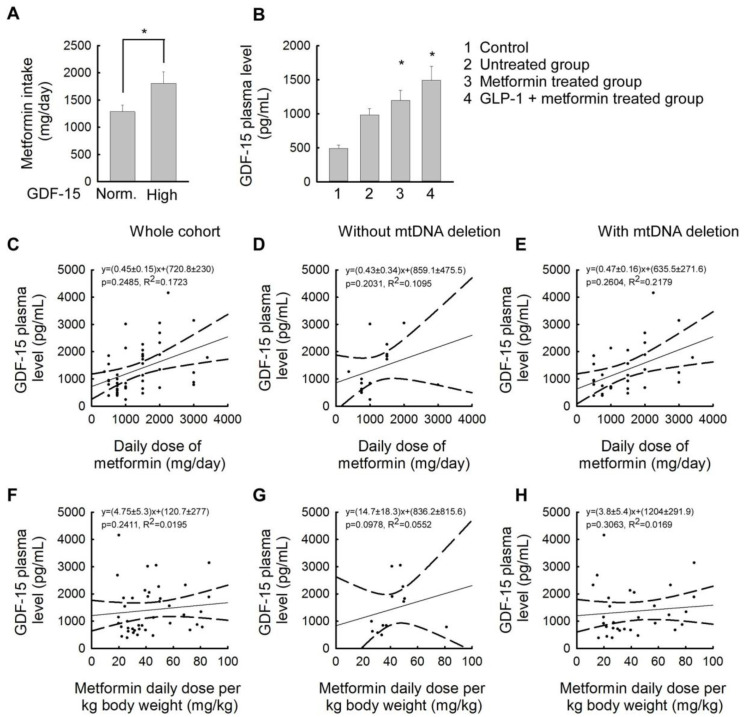
Correlations between the plasma GDF-15 levels and the dose of the administered daily metformin treatment. (**A**) Mean daily metformin intake (expressed as mg/day) in the subgroups of patients with normal (N = 40) and elevated plasma GDF-15 levels (N = 9), respectively (*: *p* < 0.05, Mann–Whitney U). (**B**) Mean plasma GDF-15 levels (expressed as pg/mL) in the healthy controls (N = 41) (1); in the subgroup of patients treated with vitamins and supplements only (N = 32) (2); in the subgroup of patients with metformin-only treatment (N = 26) (3); and in the subgroup of patients of combined metformin and GLP-1 receptor agonist treatment (N = 23) (*: *p* < 0.05, Kruskall–Wallis Dunn). (**C**) Correlation between plasma GDF-15 levels (expressed as pg/mL) and the mean daily metformin intake (expressed as mg/day) in the whole metformin-treated patient subgroup (R^2^: 0.1723) (N = 49) (4). (**D**) Correlation between plasma GDF-15 levels (expressed as pg/mL) and the mean daily metformin intake (expressed as mg/day) in the subgroup of patients without mtDNA deletions (R^2^: 0.1019) (N = 17). (**E**) Correlation between plasma GDF-15 levels (expressed as pg/mL) and the mean daily metformin intake (expressed as mg/day) in the subgroup of patients with mtDNA deletions (R^2^: 0.2172) (N = 32). (**F**) Correlation between plasma GDF-15 levels (expressed as pg/mL) and the metformin daily dose per kg body weight (expressed as mg/day) in the whole metformin-treated patient subgroup (R^2^: 0.0195) (N = 49). (**G**) Correlation between plasma GDF-15 levels (expressed as pg/mL) and the metformin daily dose per kg body weight (expressed as mg/day) in patients without mtDNA deletions (R^2^: 0.0553) (N = 17). (**H**) Correlation between plasma GDF-15 levels (expressed as pg/mL) and the metformin daily dose per kg body weight (expressed as mg/day) in patients with mtDNA deletions (R^2^: 0.0169) (N = 32).

In this section, we compared different treatments for insulin resistance in relation to patients’ plasma GDF-15 levels. The most commonly used drug in clinical practice for the treatment of insulin resistance is metformin. Of the patients studied, 32 patients received only vitamin and dietary supplementation, 26 patients required metformin treatment, and 23 patients received combined metformin and glucagon-like peptide-1 (GLP-1) receptor agonist therapy (liraglutide or semaglutide subcutaneous injections). Examining the daily metformin requirement of patients, we found that the subgroup with elevated GDF-15 levels had significantly higher metformin intake than patients with normal GDF-15 levels. (patients with normal GDF-15 levels: 1284.14 ± 786.304 mg/day; patients with elevated GDF-15 levels: 1805.56 ± 634.648 mg/day (*p* < 0.05)) (Figure 4A). In each treatment subgroup, GDF-15 levels showed a significant increase compared to the control. The group of patients with the lowest GDF-15 levels was the untreated group taking only vitamins and dietary supplements (untreated group: 982.23 ± 484.249 pg/mL; control group: 572.82 ± 82.76 pg/mL (*p* < 0.05)). Patients with the highest GDF-15 levels were treated with a combination of metformin and a GLP-1 receptor agonist (Figure 4B). In our patient group, a linear association between the daily dose of metformin treatment and plasma GDF-15 levels was observed (Figure 4C). This phenomenon was equally evident in the presence and absence of mtDNA deletions (Figure 4D,E). When considering the dose of metformin in mg/kg bw/day, the increase in plasma GDF-15 levels was less linear in the whole patient group and the subgroup of patients with mtDNA deletions than in the subgroup of patients without mtDNA deletions (Figure 4F,H).

## 3. Discussion

This study aimed to identify the role of GDF-15 and mtDNA deletions as biomarkers of mitochondrial dysfunction, which are suspected to be an underlying contributor to the pathomechanisms of insulin resistance and PCOS.

Similar to the literature published, our current analysis revealed that plasma GDF-15 levels were significantly higher in patients with IR only or IR-PCOS than in the age-matched healthy control group (Figure 1A). Previously, elevated levels of GDF-15 have been linked to several pathological conditions, such as atherosclerosis [18,19], chronic heart failure [20,21,22], chronic kidney disease [24], diabetes mellitus and insulin resistance [14,15], type 1 diabetes [25], several autoimmune disorders [26,27], several different types of cancer [28,29], cancer cachexia [30,31], severe primary mitochondrial diseases [32,33], pulmonary diseases [34,35,36], and neurodegenerative disorders (Alzheimer’s disease, cerebral stroke, and Parkinson’s disease) [37,38]. The role of elevated levels of GDF-15 in a group of women of childbearing age has previously been investigated both in plasma and in peritoneal fluid samples of patients with endometriosis and PCOS, where the level of this parameter was also elevated [39,40].

The presence of mtDNA deletions is a sensitive marker of mitochondrial dysfunction and its association with several pathological conditions is well established in the literature, such as neurodegenerative diseases, nuclear DNA (nDNA)-encoded mitochondrial diseases, and many metabolic diseases such as insulin resistance and diabetes mellitus [9,41]. According to recent literature, the presence of mtDNA deletions is now considered a potential DNA-based biomarker in cancer research. In cancer, the prevalence of mtDNA deletions is higher, mainly due to increased oxidative stress [15]. Among our patients, the presence of mtDNA deletions was confirmed in 50 cases (9 cases with single mtDNA deletion and 41 cases with multiple mtDNA deletions). Therefore, we investigated how the presence or absence of mtDNA deletions affects the plasma level of GDF-15. In our cases, no significant difference in plasma GDF-15 levels was confirmed according to the presence or absence of mtDNA deletions at the time of sampling. (Figure 1B). In addition, mtDNA deletions increase oxidative stress, which also causes beta-cell apoptosis. This mechanism has also been observed in type 2 diabetes mellitus resulting from insulin resistance [42]. However, the percentage of mtDNA deletions present in patients with normal and high plasma GDF-15 levels showed a very similar percentage distribution (43% vs. 45% without mtDNA deletions, compared with 57% vs. 55% with mtDNA deletions), thus no association between the GDF-15 biomarker and mtDNA deletion was detected in the studied patient group. In our investigated cases, we found a linear association between GDF-15 plasma levels and patient age (Figure 1C). This observation is consistent with existing literature that indicates that GDF-15 is one of the most highly regulated proteins during aging [43,44]. Our results suggest that GDF-15 is a biological aging clock parameter. It is well known that advancing age plays a role in the development of carbohydrate metabolism disorders [45,46]. The correlation between age and BMI can also be detected in our patients. In cases with mtDNA deletion, this appears to be slightly accelerated, indicating that weight gain occurs earlier (Figure 3). Therefore, in conclusion, carbohydrate metabolism disorders can also be considered multisystemic diseases due to aging, which is accelerated by mitochondrial dysfunction.

Among the investigated patients, the HOMA index and insulin levels at 60 and 120 min of insulin load were significantly higher in patients with elevated GDF-15 levels (Figure 2B). Karczewska-Kupczewska et al. investigated patients with anorexia nervosa and obesity and found that hyperinsulinemia resulted in increased serum levels of GDF-15 [47]. In addition to glucose, insulin is one of the main regulatory molecules of GDF-15, which explains its elevated levels in IR and T2DM [47,48]. In such cases, an inflammatory state is also present due to the enlarged fat mass, which in PCOS patients increases IR and hyperinsulinemia, contributing to the vicious circle of the disease [49]. In our study, 11 of the 12 patients (91.5%) with high plasma GDF-15 levels had a BMI above 25 kg/m^2^, which may also support the conclusion that these patients have a more severe metabolic disorder. Of the 12 patients with elevated GDF-15 plasma levels, 3 were treated with metformin alone, while another 7 were treated with a combination of metformin and a GLP-1 analog. In their case, the daily dose of metformin was significantly higher, which also suggested a more advanced carbohydrate metabolism disorder.

The subgrouping of our patients into BMI categories reveals that in the obese (BMI > 30 kg/m^2^) subgroup, both the plasma GDF-15 levels and the percentage of mtDNA deletion are significantly increased (Figure 3A,B). Based on our current knowledge, we cannot distinguish whether this observed phenomenon is a consequence of high plasma GDF-15 levels or a result of mitochondrial dysfunction. The receptor of GDF-15, the glial cell line-derived neurotrophic factor (GDNF) family receptor alpha-like (GFRAL) receptor, is localized in the area postrema of the brainstem [50]. It is involved in regulating body weight, food intake, and metabolism. In a GFRAL knockout mouse model the animals did not exhibit GDF-15 effects, whereas wild-type mice showed GDF-15 mediated effects via the GFRAL receptor, which resulted in reduced food intake and thus weight loss [51,52]. The GDF-15-GFRAL signaling pathway resulted in improved glucose concentrations in mice. This suggests that GDF-15 induces anorexigenic effects via the GFRAL receptor in the brainstem [50,53]. Another study has also investigated the effects of GDF-15 on food intake and energy expenditure, with similar results [54]. Chemotherapeutic administration increased GDF-15 levels in wild-type mice, resulting in a decrease in body weight, but this was not detected in GFRAL knockout animals. This effect of GDF-15 may be associated with weight loss in several chronic human diseases [55]. In rodent models, GDF-15 has been shown to complex with a coreceptor; the transmembrane tyrosine kinase molecule showed rearrangement during transfection, upon binding to its receptor, resulting in activation of the intracellular signaling pathway of GDF-15 [48,49]. Furthermore, the link between GDF-15 and weight regulation was suggested when it was found that elevated bloodstream levels of GDF-15 were associated with weight loss in individuals with advanced prostate cancer [16]. Animal studies further support this idea, as increased expression of GDF-15 resulted in leaner body composition, reduced food intake, and other beneficial changes in metabolic factors [50]. These results suggest that recombinant GDF-15 protein may be a promising therapeutic approach for the treatment of obesity and type 2 diabetes [54,56].

Recent literature data suggest that renal-specific knockout of GDF-15 expression and AP-specific knockout of GFRAL expression neutralize the ability of metformin to reduce food intake and weight gain. Altogether, this implicates the kidney as a target for metformin to regulate energy homeostasis via the renal GDF-15-dependent area postrema axis [56,57].

The patients with elevated GDF-15 plasma levels in our study had significantly higher metformin requirements than those with normal GDF-15 levels (Figure 4A). This may partly be because 3 of the 12 patients with elevated GDF-15 levels were treated with metformin alone, while another 7 cases were treated with a combination of metformin and a GLP-1 analog. It is well known from the literature that metformin treatment can also increase GDF-15 levels [58]. This is explained by metformin being an exogenous GDF-15 secretagogue whose main site of GDF-15-releasing action is in the colon [58]. Comparing the treatment approaches used in our investigated patients, it could be seen that the group of patients with the lowest GDF-15 levels were treated with only vitamin and dietary supplementation, while the patients with the highest GDF-15 values were treated with a combination of metformin and a GLP-1 receptor agonist (Figure 4B). According to the literature, GDF-15 levels are not affected by GLP-1 receptor agonists [53,59]; therefore, the increased GDF-15 plasma levels observed in our investigated cases might be related to the severity of insulin resistance. The direct correlation between plasma levels of GDF-15 and the dose of metformin taken also highlights the process of treating those with higher doses of metformin who had more marked metabolic abnormalities [57]. In our study, we could also show a linear association between the daily dose of metformin treatment and plasma GDF-15 levels (Figure 4C), which was equally evident in the presence and absence of mtDNA deletions (Figure 4D,E). When considering the dose of metformin in mg/kg bw/day, the increase in plasma GDF-15 levels was less linear in the whole patient group and the subgroup of patients with mtDNA deletions than in the subgroup of patients without mtDNA deletions (Figure 4F,H). In patients with an absence of mtDNA deletions, the linear association between GDF-15 plasma levels and daily dose of metformin in kg body weight is significantly stronger than in the subgroup with mtDNA deletions (Figure 4 G,H). Since metformin is well known in the literature to reduce visceral fat [60,61], this could be explained by the possibility that in patients without mitochondrial dysfunction, metformin acts mainly in visceral adipose tissue. This may be supported by evidence from myopathological examination of patients with primary mitochondrial myopathy, which often shows a marked accumulation of glycogen and lipids in muscle tissue samples, as well as lipid vacuolization [62,63]. This suggests that in patients with mitochondrial dysfunction, metformin may also affect intracellular lipid accumulation in tissues other than visceral adipose tissue. In the context of the above, it should be considered that in the future, the presence or absence of mitochondrial deletion could be taken into consideration when calculating metformin requirements.

The value of GDF-15 as a marker of biological age was further supported by a recent study that reported that GDF-15 is expressed to a greater extent in more rapidly aging subjects, such as Down’s syndrome patients, than in their siblings of similar age [62].

Therefore, based on our study, we conclude that elevated levels of GDF-15 and the presence of mitochondrial dysfunction may be a consequence of carbohydrate metabolism disorders in patients and may predict accelerated aging.

## 4. Materials and Methods

### 4.1. Cohort of the Study

The study included female patients treated for polycystic ovary syndrome, insulin resistance, and/or infertility at the Department of Obstetrics and Gynecology, Semmelweis University, Budapest, and having symptoms or complaints affecting several organ systems at the same time; and female patients with diagnosed mitochondrial disease having polycystic ovary syndrome and/or insulin resistance, treated at the Institute of Genomic Medicine and Rare Diseases, Semmelweis University, Budapest. Patients were selected from the Polycystic Ovary Syndrome, Mitochondrial Dysfunction, Obesity, Insulin Resistance, Infertility (POMODORI) Cohort (ClinicalTrials.gov Identifier: NCT06167135) and NEPSYBANK of the Institute of Genomic Medicine and Rare Disorders at Semmelweis University. Patient selection and genetic and biochemical tests were conducted between 2022 and 2024. Written informed consent was obtained from the patients before the sample collection and molecular genetic testing. The inclusion of patients in the present study was voluntary. The clinical status of patients and healthy control volunteers was assessed by a detailed clinical questionnaire and physical examination. The study was approved by the Hungarian National Center for Public Health Ethics (15672-6/2022/ECIG). Patients and controls enrolling in the study underwent pretest clinical genetic counseling. Molecular genetic analysis was performed for research purposes in all patients. Since plasma GDF-15 levels are known to be elevated during pregnancy, patients who were later found to be pregnant at the time of sampling were excluded from the present study. In the control group, the presence of insulin resistance, polycystic ovary syndrome, and other metabolic diseases were excluded, and all control subjects had a BMI within the normal range (20–25 kg/m^2^).

Our study included 81 patients, either with IR alone (N = 62), or with IR and PCOS simultaneously (N = 19). The mean age of patients in the study was 35.38 ± 6 (CI 95: 34.23–36.53) years; patients were aged between 20 and 45 years. The control group consisted of 41 women aged between 19 and 42 years, with a mean age of 29.82 ± 5.23 (CI 95: 28.15–31.45) years. All controls were excluded from the possibility of pregnancy at the time of sampling.

The routine laboratory parameters of the female patients included in the study were also collected: oral glucose tolerance test results (fasting, 60th-minute, and 120th-minute serum glucose and insulin levels), and HOMA index. Based on BMI and required therapy the following BMI subgroups were established: patients with BMI < 20 kg/m^2^ (N = 8); patients BMI: 20–24.9 kg/m^2^ (N = 25); patients with BMI: 25–29.9 kg/m^2^ (N = 22); patients with BMI: 30–34.9 kg/m^2^ (N = 13); and patients with BMI: >35 kg/m^2^ (N = 22).

### 4.2. Molecular Genetic Analysis

#### 4.2.1. Sample Collection and DNA Analysis

For sample collection, the DNA and plasma samples of the patients were taken at the same time. For plasma samples, special care was taken to store them at −80 °C within 1 h after sampling.

DNA isolation was performed from blood and urine epithelial cells. Based on literature data, urine epithelial cells may be a good alternative to muscle biopsy for mtDNA analysis, and therefore both tissues were used for this purpose in our patients and control subjects [63]. For DNA isolation, we used DNeasy^®^ Blood & Tissue Kits (QIAGEN GmbH, Hilden, Germany) according to the manufacturer’s instructions. For urine samples, isolation was performed from 100 mL of fresh urine. The samples were first centrifuged at 1000× *g* for 10 min; afterwards the pellet was washed with PBS and again centrifuged at 1000× *g* for 10 min. DNA isolation was performed from the resulting cell pellet using a tissue DNA isolation kit (QIAGEN GmbH, Hilden, Germany). DNA concentration was measured by UV spectrophotometer at 260 nm absorbance. The degree of purity was determined by the ratio of the 260 nm and 280 nm absorbance values.

#### 4.2.2. Analysis of mtDNA Deletions

In all blood and urine epithelial samples, the mtDNA deletion test were performed. The 4977 bp ‘common’ and multiple deletions of mitochondrial DNA were analyzed by long-range PCR. PCR was performed with 1-unit Phusion DNA polymerase (Thermo Fisher Scientific Inc., Waltham, MA, USA), GC-rich reaction buffer (Phusion GC Reaction Buffer), 200 µM dNPT 200 pM forward and reverse primer (Fw: 5′-3′ TAAAAATCTTTGAAATAGGGC, Rev: 5′-3′ CGGATACAGTTTTCACTTTAGCT), 30 ng DNA template, and nuclease-free water depending on DNA concentration, in a 20 µL reaction volume. The PCR program used was (1) initial denaturation: 98 °C 30 s; (2) 35 cycles of 98 °C 10 s, 63 °C 10 s, 72 °C 3 min; (3) final synthesis: 72 °C 7 min; (4) hold at 4 °C. The resulting fragments were separated on a 1% high-resolution agarose gel, and the PCR product bands were then sized relative to a 1 kb molecular weight marker, and the heteroplasmy ratio (ratio of wild-type to mutant mtDNA) was determined using Quantity One® 1-D Analysis Software (4.6.3 version) (Bio-Rad Imaging Systems, Hercules, CA, USA) ImageJ 1.54i software. In the case of our investigated patients, we examined mtDNA deletion in DNA samples isolated from both blood and urine epithelial cell samples. The assay amplifies the 8600 nucleotide-long region of mitochondrial DNA between nucleotides 8232 and 16,496 by long-range PCR. An mtDNA deletion was considered to be positive if in addition to the wild-type band, other smaller DNA fragment(s) were detected.

#### 4.2.3. Measurement of GDF-15 Plasma Level

GDF-15 was detected using the GDF-15 Human ELISA Kit according to the manufacturer’s instructions (Thermo Fisher Scientific, Catalog # BMS2258). Briefly, a 96-well ELISA plate was coated with primary antibody, activated, and then plasma samples were ligated and incubated. The unbound plasma was then washed and secondary antibodies were added. First, a biotinylated anti-GDF-15 antibody was bound, followed by washing. HRP-conjugated streptavidin was then bound and the substrate (TMB) was added to the solution, resulting in a blue color. Finally, the Stop reagent was added to give a yellow color and the intensity was read at 450 nm. To establish the GDF-15 values, we used as a reference range a meta-analysis published in 2022, involving approximately 20,000 people, which determined the cutoff value for GDF-15 by age group and sex [64].

### 4.3. Statistical Analysis

Generally, data are presented as the mean ± SEM. Pairs of groups were compared using the Mann–Whitney U-test, paired *t*-test and multiple groups were compared with Kruskal–Wallis Dunn multiple comparison analysis. Pearson’s and Spearman’s correlation analyses were conducted in Python, using the SciPy library’s scipy.stats.pearsonr and scipy.stats.spearmanr functions. Pearson’s correlation test was used with linear and non-linear regression models for correlational and predictive analysis. Spearman’s correlation test was used to explore monotonous but non-linear correlations, but none were significant. Pearson’s correlation analyses were visualized using linear regression. Percentages were compared using the chi-square test. Probability (*p*) < 0.05 was considered statistically significant.

## 5. Conclusions

Based on the studies we have completed so far, we have found elevated levels of the biomarker GDF-15 in 14.8% of the patients we have studied. The mean GDF-15 plasma levels were significantly higher in patients than in controls, and GDF-15 levels were also correlated with age. Dividing the study patient group into BMI ranges, the extremely obese subgroup also had higher plasma levels of GDF-15 and a higher prevalence of mtDNA deletion. In the patient group we studied, elevated GDF-15 plasma levels were associated with reactive hyperinsulinemia. The presence of mitochondrial dysfunction was mainly observed in the insulin-resistant subgroup. Correlations of the plasma GDF-15 levels with age, body mass index, and the metformin daily dose showed an increase in our study cohort. The direct correlation observed between plasma levels of GDF-15 and the dosage of metformin underscores the strategy of administering higher doses of metformin to individuals exhibiting more pronounced metabolic abnormalities.

Elevated levels of GDF-15 and the presence of mitochondrial dysfunction may be a consequence of carbohydrate metabolism disorders in patients and may be predictive of accelerated aging. From our results, we conclude that mitochondrial dysfunction may contribute to disease development at a significantly higher rate in the IR-only subgroup than in patients with IR-PCOS. The increased GDF-15 plasma levels observed in our investigated cases might be related to the severity of insulin resistance. In summary, elevated levels of GDF-15 and the presence of mitochondrial DNA deletions may be a consequence of carbohydrate metabolism disorders in patients and thus a predictor of the process of accelerated aging.

Aging biology is a promising field of research that can yield dual-purpose pathways and protein targets. These targets may impact multiple diseases while also retarding or possibly reversing age-associated processes. One widely used approach to classify the mechanisms driving aging is the hallmarks of aging. Recent analysis highlighted the partially overlapping features between aging and diseases. The GDF-15 is one example of such an overlap.

## 6. Strength and Limitations

In this study, the plasma levels of GDF-15 and the presence or absence of mtDNA deletions were first analyzed as potential biomarkers of mitochondrial dysfunction underlying insulin resistance, polycystic ovary syndrome, and their associated infertility. Patients in the study group showed a significant increase in plasma GDF-15 levels and mtDNA deletion rate compared to healthy controls. This confirms the role of these two parameters as easily detectable and useful biomarkers to assess the severity of these clinical features.

In addition, these biomarkers can also be used to monitor patients’ current pharmacological therapy, as they change in parallel with the severity of insulin resistance. Thus, in moderate IR, metformin or, in more severe cases, metformin and GLP-1 agonist treatment should be used as the patient’s pharmacotherapy, thus helping to ensure proper carbohydrate metabolism and contributing to the success of childbearing.

A limitation of our study may be that we were only able to perform this comprehensive study on a relatively small number of cases, so future studies on a larger group of patients and other mitochondrial biomarkers may be needed.

The mean age of healthy volunteer controls differs by 5.56 years from the mean age of the investigated patients. Although this may slightly influence the results because GDF-15 increases with age, the difference in the mean GDF-15 value between the control group and the patient group in our study is significantly larger than the difference in the patient group at 5 years, and therefore we consider our results valid.

## Data Availability

The data presented in this study are available on request from the corresponding author.

## Supplementary Materials

The following supporting information can be downloaded at: https://www.mdpi.com/article/10.3390/ijms252010916/s1.
