# Supplementary material for: GDF-15 and mtDNA Deletions Are Useful Biomarkers of Mitochondrial Dysfunction in Insulin Resistance and PCOS
Source: Int J Mol Sci. 2024 Oct 10;25(20):10916. doi: 10.3390/ijms252010916 (PMC11507876; doi:10.3390/ijms252010916)
Supplement: Supplementary file 1 [file ijms-25-10916-s001.zip › Supplementary Materials/Supplementary Figures with Legends.docx]

**Supplementary Materials:**


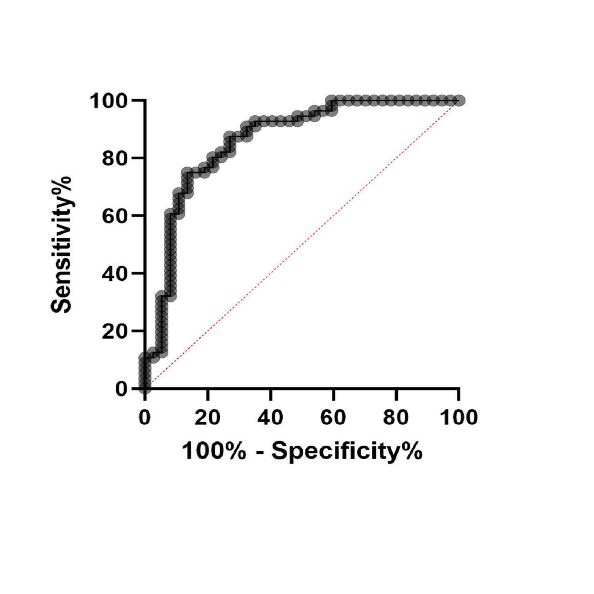


**Figure S1:** The receiver operating characteristic (ROC) curve analysis based on the GDF-15 results of our patients and the healthy control group.


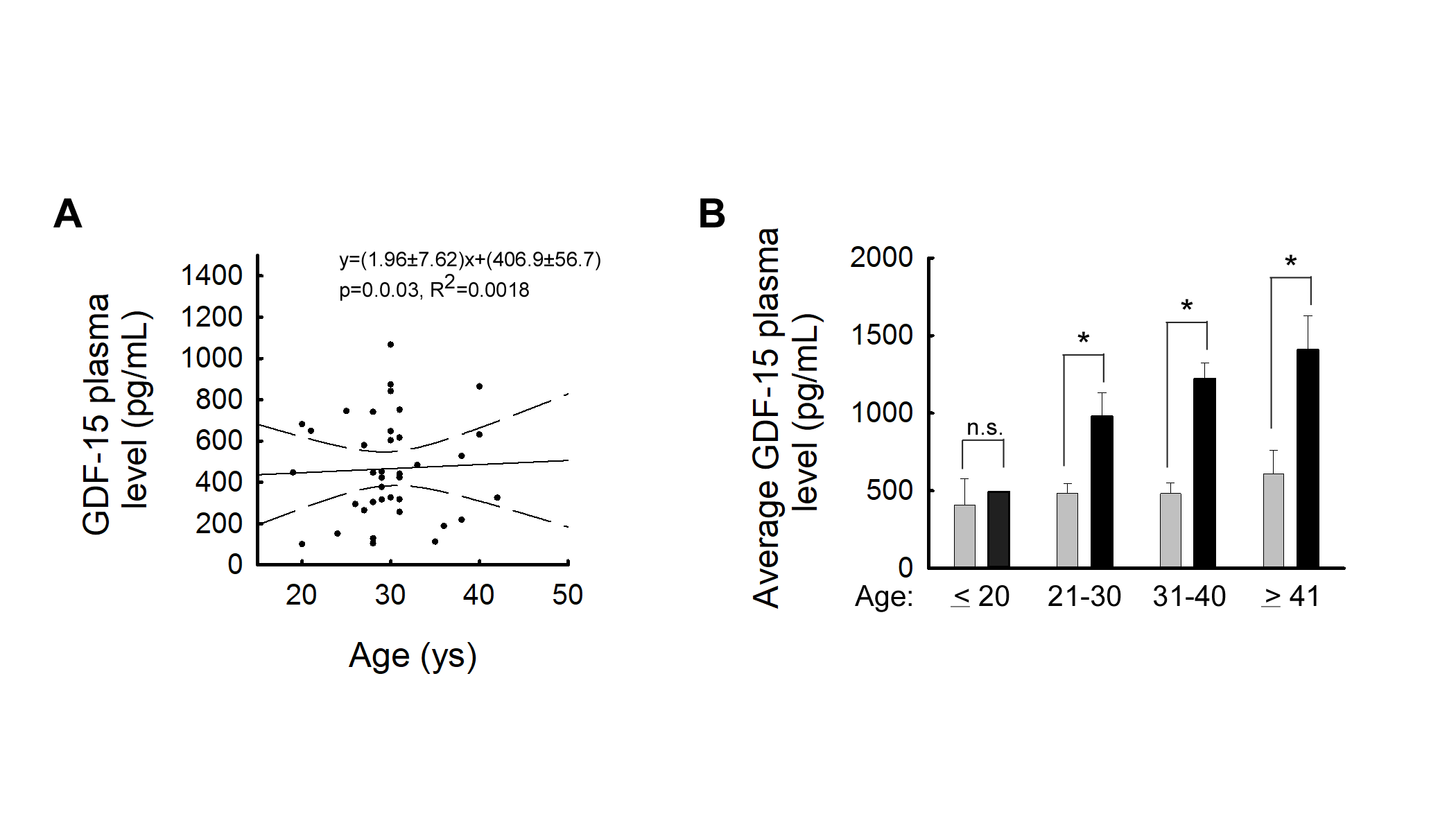


**Figure S2:** Correlation of plasma GDF-15 levels and the age at examination **(A)** for the total study patient group **(B)** comparison of the study group and the control group by age range in terms pf plasma GDF-15 levels.
