# Supplementary figures and images for: GDF-15 and mtDNA Deletions Are Useful Biomarkers of Mitochondrial Dysfunction in Insulin Resistance and PCOS
Source: Int J Mol Sci. 2024 Oct 10;25(20):10916. doi: 10.3390/ijms252010916 (PMC11507876; doi:10.3390/ijms252010916)

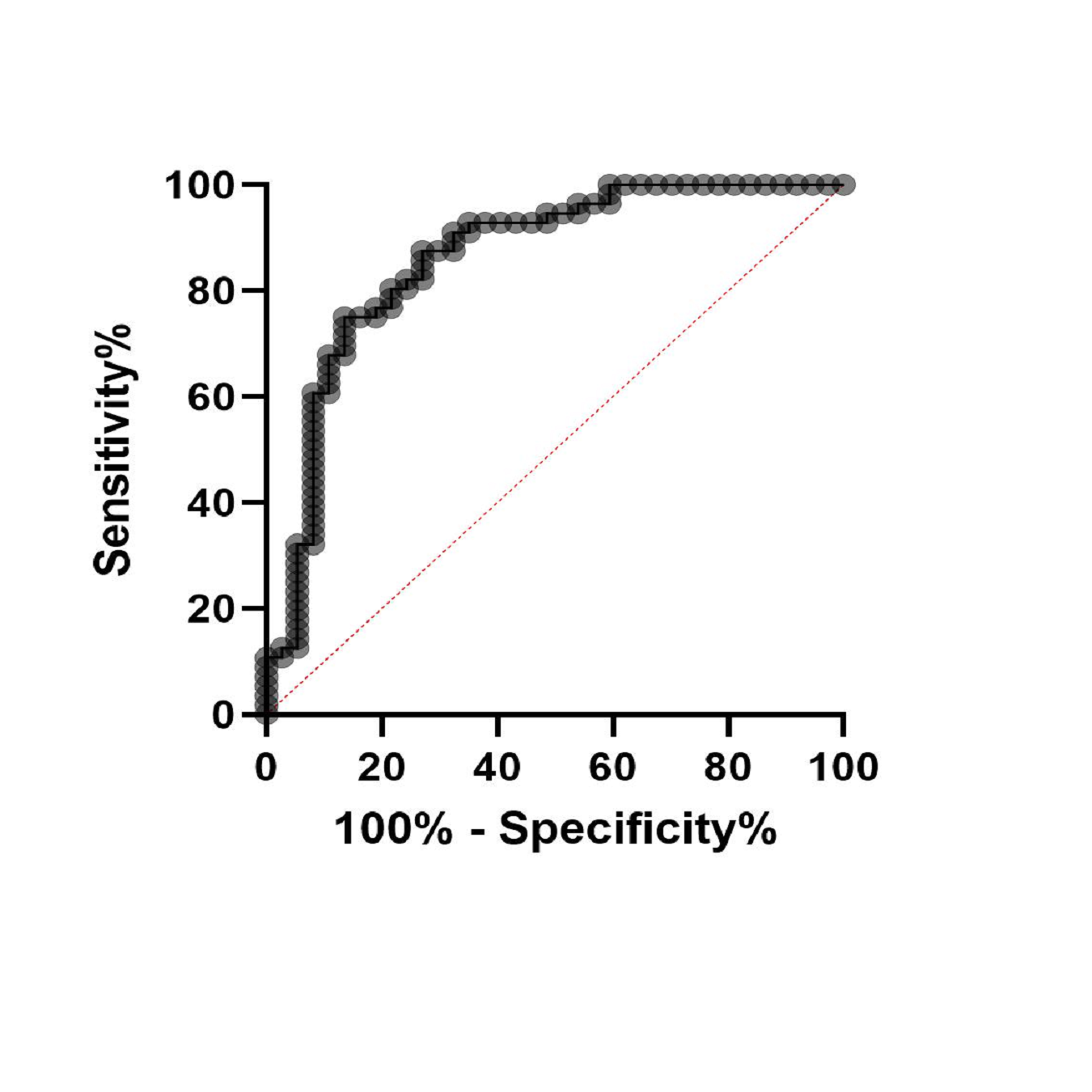

Supplement: Supplementary file 1 [file ijms-25-10916-s001.zip › Supplementary Materials/Figure S1.tif]

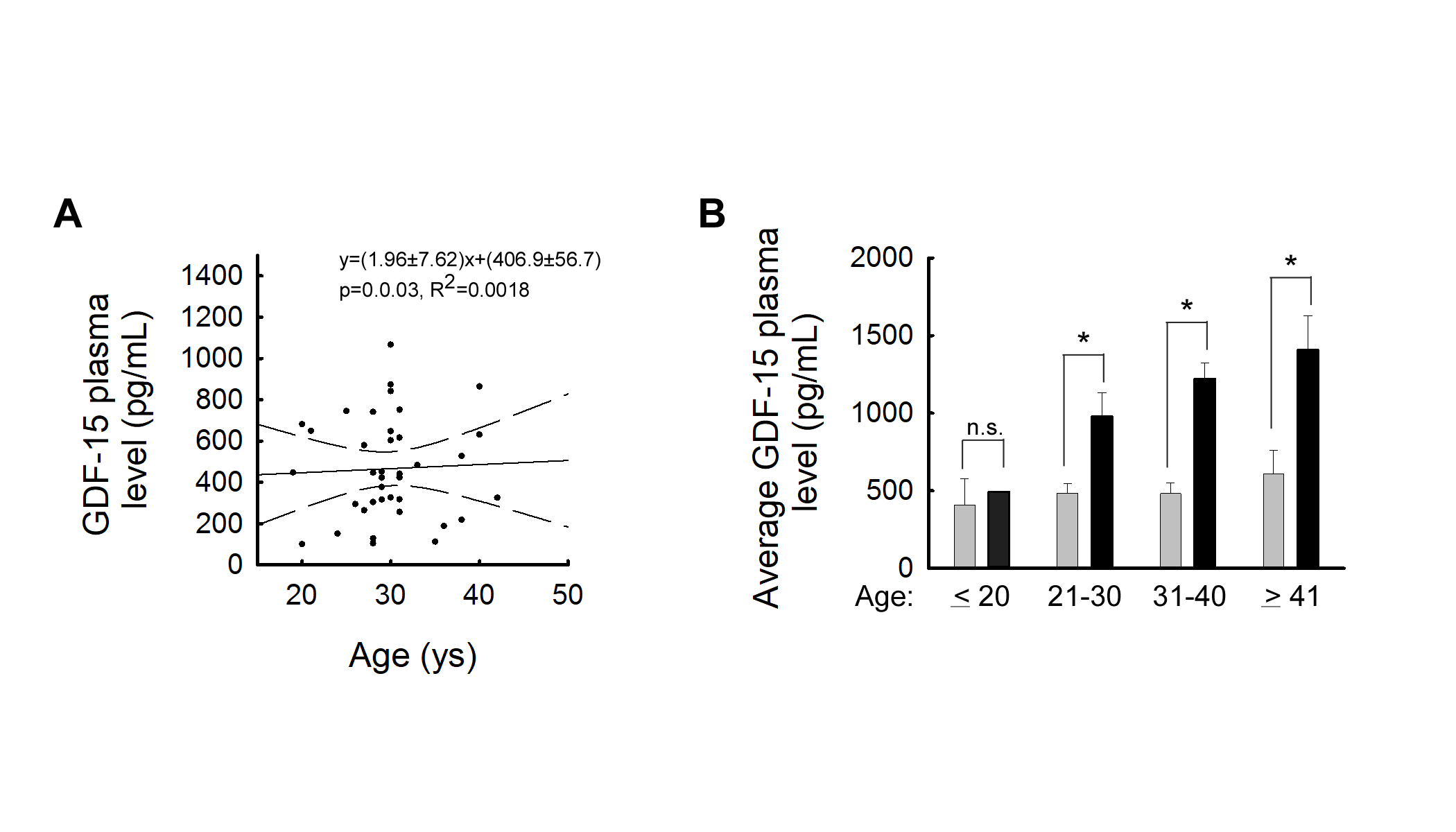

Supplement: Supplementary file 1 [file ijms-25-10916-s001.zip › Supplementary Materials/Figure S2.tif]
